# Evaluation of Hypoxia Markers in Critically Ill Patients Categorized by Their Burden of Organ Dysfunction: A Novel Approach to Detect Pathophysiological and Clinical Relevance in a Secondary Analysis of a Prospective Observational Study

**DOI:** 10.3390/ijms26020659

**Published:** 2025-01-14

**Authors:** Franz-Simon Centner, Kathrin Brohm, Sonani Mindt, Evelyn Jaeger, Bianka Hahn, Tanja Fuderer, Holger A. Lindner, Verena Schneider-Lindner, Joerg Krebs, Michael Neumaier, Manfred Thiel, Jochen J. Schoettler

**Affiliations:** 1 Department of Anesthesiology, Surgical Intensive Care Medicine and Pain Medicine, Medical Faculty Mannheim, University Medical Center Mannheim, University of Heidelberg, Theodor-Kutzer-Ufer 1-3, 68167 Mannheim, Germany; franz-simon.centner@umm.de (F.-S.C.); jochen.schoettler@umm.de (J.J.S.); 2 Merck KGaA (SQ-Animal Affairs), Frankfurterstrasse 250, 64293 Darmstadt, Germany; 3 Institute for Clinical Chemistry, Medical Faculty Mannheim, University Medical Center Mannheim, University of Heidelberg, Theodor-Kutzer-Ufer 1-3, 68167 Mannheim, Germany; 4 Institute for Laboratory and Transfusion Medicine, Hospital Passau, Innstrasse 76, 94032 Passau, Germany

**Keywords:** organ dysfunction, hypoxia, sequential organ failure assessment score, lactate, s-adenosylhomocysteine, mortality prediction, critically ill patients

## Abstract

In critically ill patients, compromised microcirculation causes tissue hypoxia, organ failure, and death. These pathophysiological processes occur particularly in patients with high illness severity, so reliable hypoxia biomarkers should reflect this in their occurrence. This secondary analysis of a prospective study categorized patients by their burden of organ dysfunction (BOD) using the cohort’s median initial sequential organ failure assessment (SOFA) score of 8 as a cutoff. The kinetic parameters of the hypoxia markers lactate and S-adenosylhomocysteine (SAH) were analyzed for correlation with organ dysfunction severity and mortality prediction. In low BOD patients, neither marker correlated with SOFA. In high BOD patients, lactate showed a moderate correlation and SAH showed a strong correlation. Lactate correlated with organ dysfunction in survivors but not in non-survivors, while SAH correlated strongly in non-survivors but not in survivors. In univariate logistic regression, lactate predicted mortality moderately in low BOD (areas under the receiver operating characteristic curves (AUROCs) 0.7–0.8) but poorly in high BOD patients (AUROCs 0.5–0.7). SAH’s prediction improved from poor to excellent (AUROCs 0.8–0.9) with higher BOD. Thus, SAH appears superior to lactate in the detection of organ dysfunction severity and mortality prediction in high BOD patients.

## 1. Introduction

The development of multiple organ dysfunction syndrome (MODS) is one of the most critical clinical events in critically ill patients, as it is directly linked to morbidity and mortality [1,2]. Although the full spectrum of pathogenetic mechanisms leading to organ failure is still not fully understood, impaired microcirculation and consecutive tissue hypoxia play an important role [3,4,5]. Numerous infectious and non-infectious conditions are known to cause inflammatory damage to microcirculation [3] and mitochondrial dysfunction [6]. This results in comprised perfusion-dependent oxygen delivery and subsequent energetic failure, leading to multiple organ dysfunction and failure [3].

The lack of a reliable marker to assess cellular hypoxia remains a major challenge in intensive care medicine [7]. From a clinical perspective, one of the most intriguing questions is the prediction of the clinical course of organ dysfunction in a patient upon ICU admission, which is of special interest in the most severely ill subpopulation [1,7,8]. With this study, we aimed to contribute to the early biomarker-supported prediction regarding the clinical course of organ dysfunction and mortality in critically ill patients underlying the following considerations:

Currently, lactate is used as a marker of hypoxia in critically ill patients [7,9]. When analyzing serial lactate levels, mean and maximum lactate showed better performance in predicting mortality in critically ill patients with and without sepsis than single values [10,11]. Even better predictive power was achieved when lactate levels were plotted against time, calculating the area under the curve (AUC), known as the lactate area score [11,12]. By dividing the AUC by the time interval, the ‘normalized area score’ (NAS) of lactate was obtained for the respective period, which was supposed to reflect the patient’s hypoxic burden [10,11]. However, while, during exercise, hyperlactatemia clearly results from an imbalance between oxygen delivery and requirement, in critically ill patients, the genesis of hyperlactatemia is significantly more complex [7]. Lactate represents a downstream metabolic product whose origin does not always reflect insufficient organ perfusion and consequently lacks specificity for indicating microcirculatory impairment in critically ill patients [7,9,13,14,15].

In addition to its association with cardiovascular and renal diseases [16,17,18,19], S-adenosylhomocysteine (SAH) has been identified as a metabolic marker for tissue hypoxia [20,21,22]. It has recently proven useful in the early prediction of septic disease progression and mortality in critically ill patients with superior performance compared to lactate [23,24]. To further explore the prognostic value of SAH in critically ill patients, in this study, we focus on its relation to organ dysfunction. Therefore, we investigated the association of the kinetic parameters of lactate and SAH with those of the sequential organ failure assessment (SOFA) score as an established representation of organ dysfunction [25,26]. To our knowledge, this is the first study to quantify the time course of the SOFA score, calculating its normalized AUC to reflect the burden of organ dysfunction (BOD), a term introduced with this study in analogy to the burden of hypoxia captured by the NAS of lactate and SAH [10,11].

Pathogenetic processes like inflammation, microcirculatory injury, and hypoxic and dysoxic energetic failure are thought to precede organ dysfunction, leading to an increase in the risk of death [3,27,28,29]. There is increasing evidence that microcirculatory compensation mechanisms are impaired by the severity of illness [4,5]. Consequently, it was suggested that studies related to the pathophysiology of microcirculation should be conducted specifically in critically ill patients with high SOFA scores [4,5]. To address this, we categorized a cohort of critically ill patients into a group with low and a group with high BOD using the median initial SOFA score as a cutoff [5]. Metabolites indicative of energetic failure, such as lactate and SAH, are expected to be higher in patients with a higher severity of illness compared to those with a lower one.

Accordingly, we hypothesized that critically ill patients with a high BOD compared to those with a low BOD would exhibit:

- Higher levels of hypoxia markers;
- Better performance in the kinetic parameters of serially measured SOFA scores and hypoxia markers in predicting death;
- Stronger correlations between the kinetic parameters of hypoxia markers and the respective SOFA scores.

In patients with severe organ dysfunction in this study, it was SAH, not lactate, that mirrored the kinetics of organ dysfunction and enabled excellent early mortality prediction.

## 2. Results

### 2.1. Categorizing Patients Based on the Median Initial SOFA Score

**Figure 1:**
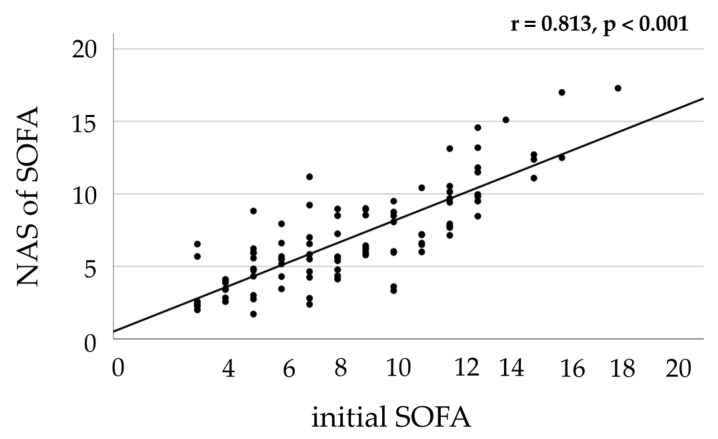
Linear regression correlation between initial SOFA scores and normalized area scores (NASs) of the SOFA. Initial SOFA scores exhibited a significant and linear correlation with the NAS of the SOFA. Each data point represents a pair of variables from an individual patient. The number of measurements used to calculate the NAS was, on average, 11 to 12 per patient assessed over a period of up to 5 days. Abbreviations: SOFA = Sequential Organ Failure Assessment.

In a previous study [23], we prospectively enrolled 99 critically ill patients who were, for the present study, retrospectively categorized based on the median SOFA score of 8 of the total cohort at study inclusion (initial, time point 1). The initial SOFA score showed a strong correlation (r = 0.813) with the NAS of the SOFA, which incorporated scores over a period of up to 5 days (Figure 1). Since the possibility of early prediction is important for clinical application, this strong correlation justified the use of the initial SOFA score for the categorization of the cohort into the low and high BOD groups.

**Figure 2:**
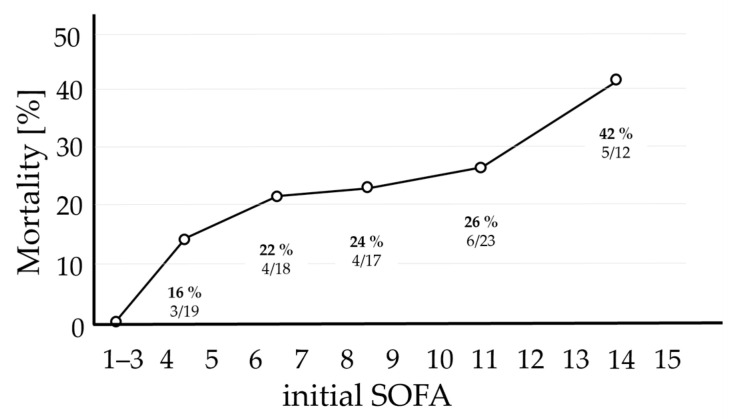
Mortality by initial SOFA score among the cohort of critically ill patients. The SOFA scores for the total cohort ranged from 1 to 19. To ensure enough patients for a meaningful calculation of mortality rates, SOFA scores were categorized into groups (1 to 3, 4 to 5, 6 to 7, 8 to 9, 10 to 12, and 13 to 15). The corresponding values of mortality rates are displayed below the number of non-survivors in relation to the total number of patients per group. There were 7 patients with SOFA scores between 1 and 3 and 3 patients with scores between 16 and 19. None from the former group and two from the latter group died, resulting in mean mortality rates of 0% and 66.6%, respectively (these rates are not shown). As expected, the mortality rates escalated with increasing SOFA scores. Abbreviations: SOFA = Sequential Organ Failure Assessment.

In total, 53 patients with initial SOFA scores less than or equal to the median (SOFA ≤ 8) were deemed to have a low BOD, and 46 patients with scores above the median (SOFA > 8) had a high BOD. For validation and comparison with previously published data [25,30,31,32], SOFA scores and their relationship with mortality are displayed in Figure 2. There was an inflection point in mortality between a SOFA score of 8 and 9 which was used as the cutoff between low and high BOD.

### 2.2. Patient Groups with Low and High BOD Show No Difference Regarding Overall Occurrence of Sepsis

**Figure 3:**
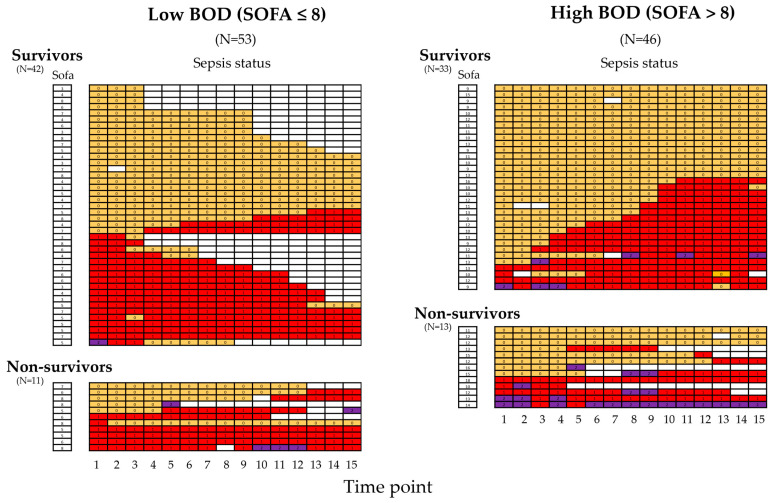
Sepsis status over time among critically ill patient groups with low and high burden of organ dysfunction (BOD). Out of the total cohort of critically ill patients (N = 99), two groups were formed based on the median initial SOFA score, which was 8. This led to the creation of a group of patients with low BOD (SOFA score ≤ 8, N = 53) and a group with high BOD (SOFA score > 8, N = 46). Additionally, patients were subdivided by mortality into survivors and non-survivors. The initial SOFA score of each patient is given in the first column to the left of each group’s spreadsheet. The sepsis status of each patient was determined at study inclusion (time point 1) and thereafter every 8 h, up to a total of 15 assessments, to determine the absence of sepsis (0, orange fill), the presence of sepsis (1, red fill), or septic shock (2, purple fill). Significant differences in initial SOFA scores were observed between patients with low and high BOD across all patients, as well as within the survivors and non-survivors subgroups (*p* < 0.001 for all, Mann–Whitney U test). No significant difference in mortality rates was found between patients with low and high BOD (chi-square 0.755, *p* = 0.387). The overall proportion of patients suffering from sepsis or septic shock, whether present at time point 1 or developing thereafter, did not differ between patients with low BOD and those with high BOD (chi-square 0.036, *p* = 0.899). This finding was consistent when tested separately among survivors and non-survivors, respectively. Abbreviations: SOFA = Sequential Organ Failure Assessment.

The group of patients with low BOD consisted of 53 patients, of whom 42 survived and 11 died (21%). The group of patients with high BOD included 46 patients with 33 survivors and 13 deaths (28%) (Figure 3). The proportion of non-survivors did not differ significantly between the low and high BOD groups (chi-square 0.755, *p* = 0.3847). All patients were classified according to sepsis-3 criteria at study inclusion and subsequently up to 14 times every 8 h over 5 days to monitor sepsis dynamics. In the low BOD group at study inclusion, 29 of 53 patients (54.7%) were not septic, 23 patients were septic (43.4%), and 1 patient had septic shock (1.9%). Of the 29 initially non-septic patients, 9 developed sepsis during the study period (27.6%), with 2 of these patients developing septic shock. In patients with high BOD, 37 of 46 patients (80.5%) were not septic, 6 patients were septic (13.0%), and 3 had septic shock (6.5%). Of the 37 initially non-septic patients, 19 developed sepsis during the study period (51.4%), with 4 of these patients developing septic shock. In patients with low BOD, the proportion of patients with initial sepsis was significantly higher than in patients with high BOD (chi-square 7.3297, *p* = 0.007). In patients with high BOD, there was a trend toward more patients developing sepsis (chi-square 3.794, *p* = 0.051). However, the overall proportion of patients experiencing sepsis during the study period did not differ between the low and high BOD groups (chi-square 0.0361, *p* = 0.899). When the overall frequency of sepsis/septic shock was analyzed separately in survivors and non-survivors, again, no difference was found between patient groups with low and high BOD.

### 2.3. Baseline and Clinical Characteristics of Study Participants

Demographic data, clinical course, primary diagnosis, comorbidities, clinical chemistry, hematology, vital signs, clinical scores, and hypoxia biomarkers were determined for all patients at study inclusion (Table 1). Variables were compared between patient groups with low BOD (SOFA ≤ 8) and high BOD (SOFA > 8) and within each group between survivors and non-survivors. Although there was no significant difference between patients with low and high BOD in terms of mortality or the occurrence of sepsis, patients with high BOD were younger, more frequently male, more often mechanically ventilated and vasopressor-dependent, and had a higher shock index and longer ICU stay. Patients with higher BOD showed more instances of polytrauma and major bleeding but less frequent sepsis and major surgery. These patients less frequently exhibited cardiac and pulmonary comorbidities, arterial hypertension, and diabetes mellitus. Kidney, liver, and pancreas function/integrity markers were significantly elevated in patients with high BOD compared to patients with low BOD. Inflammation- and infection-associated parameters were significantly more altered in patients with high BOD, indicating a higher activation and/or consumption. The need for sedation and therapeutic intervention, as assessed by RASS and TISS, was also significantly higher in these patients. Fittingly to the grouping by BOD, the SOFA score was twice as high in patients with high BOD compared to patients with low BOD. Initial plasma concentrations of lactate and SAH were significantly higher in patients with high BOD (*p* = 0.004 and 0.018, respectively; Table 1).

Comparing these respective parameters between non-survivors and survivors in patients with low BOD revealed no differences, except for the SOFA score, which was significantly higher in non-survivors. In patients with high BOD, non-survivors were generally older, had shorter ICU stays, presented more often with sepsis and less frequently with polytrauma, and had higher serum levels of creatinine and urea, as well as higher TISS-, SAPS II-, and SOFA scores compared to survivors. The initial plasma levels of lactate and SAH were significantly higher in non-survivors compared to survivors (*p* = 0.031 and <0.001, respectively; Table 1).

### 2.4. Time Course of SOFA Score, Lactate, and SAH Plasma Concentrations in Patients Grouped by BOD and Mortality

**Figure 4:**
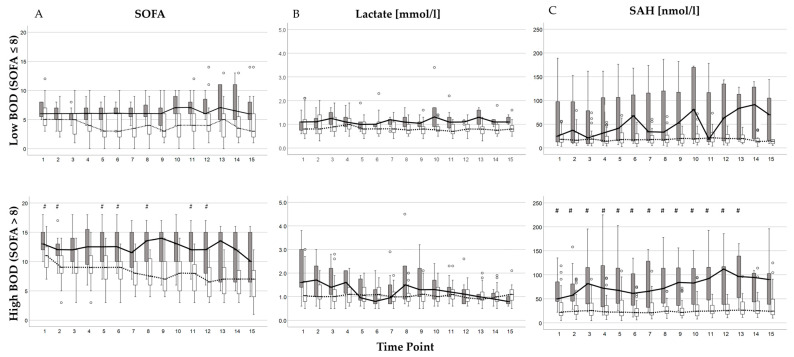
Progression of the SOFA score (**A**), lactate (**B**), and SAH plasma concentrations (**C**) in survivors and non-survivors grouped by their initial burden of organ dysfunction (BOD). The upper and lower panels show time series data for patients with a low (SOFA ≤ 8) and high (SOFA > 8) BOD, respectively. The data for non-survivors and survivors are represented in filled and empty box plots, where the top and bottom lines of the box denote the 25th and 75th percentiles, respectively. The median value is indicated by the horizontal line within the box. A line connecting these median values (solid for non-survivors, dashed for survivors) illustrates the areas under the curves of each contributing patient. Outliers, which are values greater than 1.5 times but less than 2.5 times the interquartile range, are marked with empty circles. Comparisons between non-survivors and survivors were made at each time point using the Mann–Whitney U test, with significance levels adjusted according to Bonferroni correction (#: *p* ≤ 0.003). In patients with low BOD, no significant differences were observed in SOFA scores (**A**) between non-survivors and survivors. However, in patients with high BOD, non-survivors consistently exhibited significantly higher SOFA scores compared to survivors. Throughout the study period, lactate plasma concentrations (**B**) showed no significant differences between non-survivors and survivors, regardless of the severity of organ dysfunction. In contrast, SAH (**C**) was consistently significantly higher in non-survivors than in survivors among patients with high BOD. Abbreviations: SOFA = Sequential Organ Failure Assessment; SAH = S-adenosylhomocysteine.

Because of transfer to other wards or death, the actual number of evaluated 8 h intervals was 1274. After the subtraction of missing values, the number of SOFA scores finally analyzed was 1143, 1126 blood samples for lactate, and 1248 for SAH, resulting in determination rates of 89.7%, 88.4%, and 97.9% during the study period, respectively. These measurements served to calculate the kinetic parameters to characterize the time course of BOD and the putative burden of hypoxia. The serially determined values of the SOFA score, lactate, and SAH plasma concentrations during the study period were analyzed separately in patients with low and high BOD and grouped by mortality at each time point of the study period (Figure 4). At no time point throughout the study period did patients with low BOD (Figure 4 upper panel) show significant differences between non-survivors and survivors regarding the SOFA score (Figure 4A) or plasma concentrations of lactate (Figure 4B) or SAH (Figure 4C). In patients with high BOD (Figure 4 lower panel), non-survivors showed significantly higher values than survivors for the SOFA score (Figure 4A) and plasma concentrations of SAH (Figure 4C) at time point 1. However, the level of significance for lactate (Figure 4B), as presented in Table 1, was lost due to Bonferroni correction for multiple tests. Throughout the study, significantly higher values for the SOFA score (Figure 4A, lower panel) and plasma concentration of SAH (Figure 4C, lower panel) were repetitively observed in non-survivors compared to survivors, despite correction for multiple testing. For plasma levels of lactate (Figure 4B, lower panel), no significant differences were observed between non-survivors and survivors at the subsequent time points.

### 2.5. Performance of SOFA Scores, Lactate, and SAH Plasma Concentrations in Patients with Low and High BOD for Prediction of In-Hospital Mortality

Compared to patients with low BOD, patients with high BOD exhibited significantly higher initial values and kinetic parameters (max, mean, NAS) of the SOFA score, lactate, and SAH plasma concentrations (Table 2). In patients with low BOD, initial values and kinetic parameters of the SOFA score, lactate, and SAH plasma concentrations were generally higher in non-survivors than in survivors. However, these differences did not reach statistical significance for maximum SOFA, initial lactate, and any SAH parameter. In patients with high BOD, initial values and kinetic parameters of the SOFA score, lactate, and SAH plasma concentrations were all significantly higher in non-survivors compared to survivors (Table 2).

Accordingly, in patients with low BOD, in univariate logistic regression analysis, the significant prediction of in-hospital mortality was achieved by the initial values of the SOFA score and SAH plasma concentrations, as well as the kinetic parameters of lactate and SAH, with the exception of maximum SAH (Table 3). Regarding the corresponding areas under the receiver operating characteristic curves (AUROCs), except for maximum SOFA, all SOFA score-based parameters, as well as the kinetic parameters of lactate, reached the level of significance. For SAH, none of the AUROCs were significant. Only the AUROCs of the kinetic parameters (mean and NAS) of lactate proved to be significantly greater than those for initial values.

In patients with high BOD, all parameters of the SOFA score and SAH plasma concentrations significantly predicted in-hospital mortality (Table 3). In contrast, initial values and kinetic parameters of lactate did not significantly predict in-hospital mortality, except for the NAS. The AUROCs of the kinetic parameters of the SOFA score were all above 0.8 and higher compared to the AUROC of the initial value. The AUROC of initial lactate was slightly above 0.7, while the values of kinetic parameters were all below 0.7. Conversely, the AUROC values for both the initial values and kinetic parameters of SAH were all above 0.8. Compared to the AUROC determined at baseline (0.825), the values of the kinetic parameters were even higher. However, there was no statistically significant difference between the AUROCs of the initial values and the kinetic parameters for any parameter.

**Figure 5:**
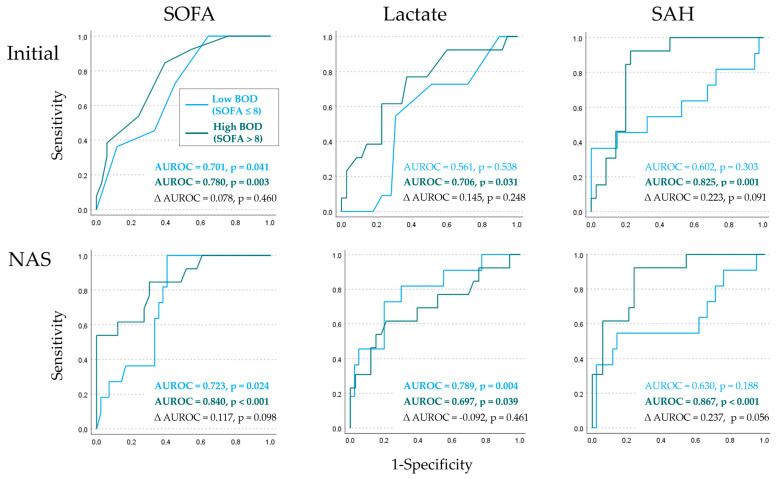
Receiver operating characteristic curves (ROC) of mortality prediction by univariate logistic regression models in patients with low and high burdens of organ dysfunction (BOD). ROC analyses were conducted for SOFA scores, lactate, and SAH plasma concentrations in two patient groups: those with low (SOFA ≤ 8) and high BOD (SOFA > 8). For clarity, only the area under the ROC curve (AUROC) values determined initially and for NAS are shown. AUROCs for initial SOFA, lactate, and SAH were all lower in patients with low BOD compared to those with high BOD, yielding positive AUROC differences between patient groups (∆ AUROC = AUROC_high BOD_ − AUROC_low BOD_). While these differences in AUROCs remained positive for the kinetic parameters of SOFA and SAH, as illustrated by NAS (for maximum and mean values, refer to Table 3), the differences in AUROCs became negative for lactate kinetic parameters due to progressively higher values in patients with low BOD compared to those with high BOD. None of the calculated AUROC differences were statistically significant, except for a trend observed for the NAS of SAH (lower right panel). Abbreviations: SOFA = Sequential Organ Failure Assessment; SAH = S-adenosylhomocysteine; NAS = normalized area score.

When only statistically significant results of both logistic regression and AUROC analyses with values greater than 0.7 (at least acceptable discrimination according to the classification by Hosmer and Lemeshow [33]) were taken into account, mortality prediction by lactate was better in patients with low BOD compared to those with high BOD. The opposite situation was observed for SAH (Table 3). When AUROC values of the SOFA score, lactate, and SAH were tested for differences between patient groups with low and high BOD, the mean and NAS of SAH showed a trend toward statistical significance (*p* = 0.064 and 0.056, Table 3). AUROCs of SOFA, lactate, and SAH along with AUROC differences are visualized for initial values and NAS in Figure 5.

### 2.6. Correlation Between Initial Values and Kinetic Parameters of Lactate and SAH Plasma Concentrations and Corresponding SOFA Scores in Patients Grouped by BOD and Mortality

In patients with low BOD, except for mean SAH, which showed a moderate correlation with mean SOFA (r < 0.5), there were no significant correlations between the initial values or kinetic parameters of lactate or SAH and the corresponding SOFA scores, regardless of whether the correlation was analyzed using linear regression (Table 4) or Spearman’s methods (Supplementary Table S1).

In contrast, in patients with high BOD, the initial values and kinetic parameters of lactate and SAH significantly correlated with the corresponding SOFA scores. While the correlations observed for lactate were of moderate strength (r < 0.5), those for SAH were strong (r > 0.5).

When patients with low BOD were subgrouped by mortality, neither in survivors nor in non-survivors were significant correlations between the initial values or kinetic parameters of lactate or SAH and the corresponding SOFA scores found.

In patients with high BOD who survived, the initial values and kinetic parameters of lactate correlated significantly with the corresponding SOFA scores, although the strength of the correlation was only moderate. Regarding SAH, only initial values correlated significantly with the initial SOFA score, while there were no significant correlations between the kinetic parameter values of SAH and the corresponding SOFA scores (Table 4 and Supplementary Table S1).

**Figure 6:**
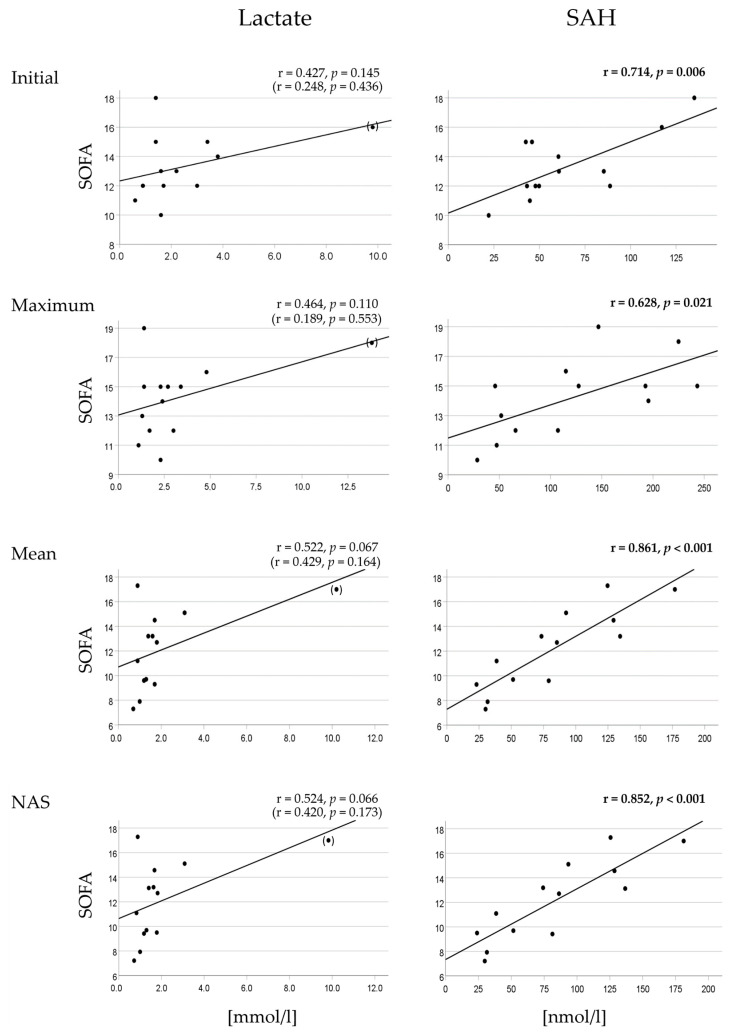
Correlations between the initial values and kinetic parameters of lactate and SAH plasma concentrations and corresponding SOFA scores in non-survivors with a high burden of organ dysfunction. While the initial values and kinetic parameters of lactate did not show a significant correlation with the corresponding SOFA scores, the SAH parameters exhibited a significant and linear correlation. When the noticeable outlier in the lactate analysis was removed, the correlations between corresponding lactate and SOFA parameters were diminished (results for linear regression coefficients and *p*-values after the removal of the outlier are given in parentheses). Each data point in a panel represents a pair of variables from an individual patient. In the panels that display the correlation of mean values or NAS with the corresponding SOFA scores, the number of measurements used to calculate the mean or NAS for a variable was, on average, 11 to 12 per patient. Abbreviations: SAH = S-adenosylhomocysteine; SOFA = Sequential Organ Failure Assessment; NAS = normalized area score.

In patients with high BOD who died, no correlations were observed for the initial values or kinetic parameters of lactate with the corresponding SOFA scores. In contrast, initial and kinetic parameter values of SAH showed strong and significant correlations with the corresponding SOFA scores (Table 4, Figure 6, and Supplementary Table S1).

## 3. Discussion

Systemic inflammation appears to be a common factor in both infectious and non-infectious conditions, leading to compromised microcirculation, energy metabolism, and hypo- and dysoxia, which can result in multiple organ failure and death [3,6]. In various diseases associated with critical illness, it has been shown that higher illness severities manifest with higher levels of inflammation and microcirculatory impairment [3,4,5,27,28,29]. Fittingly, there is increasing evidence that microcirculatory compensation mechanisms are impaired by the severity of illness independently from the underlying disease entity [3,4,5,27,28,29]. Consequently, it was suggested that studies related to the pathophysiology of microcirculation should be conducted specifically in critically ill patients with high SOFA scores [4,5].

SOFA is associated with organ dysfunction from various causes, regardless of whether they are of infectious or non-infectious origin [30], and it has proven to be a reliable predictor of mortality in diverse ICU populations [8]. Thus, while SOFA might be a clinical scoring system that can capture the uniform pathophysiology of microcirculatory impairment in severely ill patients, a reliable biomarker is missing [7]. Therefore, in this study, we chose the SOFA score as a reference for the investigation of the presumed hypoxia marker SAH and used lactate as the current standard [7,9] for comparison. A reliable hypoxia marker would be expected to exhibit behavior analogous to that of the SOFA score. Additionally, it should reflect both the severity of organ dysfunction and its correlation with mortality.

To investigate this, pursuing a similar approach as Brouwer et al. [5] for patient categorization, we classified critically ill patients into two groups using the median initial SOFA score of the cohort, which was 8, as the cutoff. In different cohorts of critically ill patients, mortality increased significantly in patients with SOFA scores of 9 or higher [30,31,32]. Also, in our cohort, there was an inflection point in mortality between a SOFA score of 8 and 9 (Figure 2). The initial SOFA scores showed strong correlations with the NAS of the SOFA, which incorporated scores over a period of up to 5 days. Since early prediction is crucial for clinical application, this strong correlation justified the use of the initial SOFA score for categorizing the cohort into low and high BOD groups. Additionally, Soo et al., when analyzing organ dysfunction in 20,000 ICU patients, concluded that the severity of organ dysfunction is usually at its worst at admission [26]. Using this approach, several hypotheses were investigated to examine the behavior of the hypoxia markers:

Hypothesis (i) anticipated higher levels of hypoxia markers in patients with high BOD compared to those with low BOD. This was confirmed by the significantly higher initial levels and kinetic parameters of lactate and SAH in patients with high BOD (Table 1 and Table 2). These results indicate that a higher BOD is associated with a greater burden of tissue hypoxia [10,11], not only at study inclusion upon ICU admission but also throughout the five-day study period. Since the overall presence of sepsis was not statistically different between the two groups, sepsis seems unlikely to account for the observed differences (Figure 3). Supporting this, the frequency of sepsis was even higher in patients with low BOD at admission (Table 1 and Figure 3). However, although the sepsis-3 criteria were designed to capture life-threatening organ dysfunction [35], there is substantial literature criticizing the increase in the SOFA score by 2 points as suboptimal for diagnosing sepsis [36,37,38,39]. For instance, a SOFA score of 6 has been identified as a potentially better diagnostic criterion in a cohort of critically ill patients [36]. This might explain the lack of difference in sepsis frequency between patients with high and low BOD in this study.

Hypothesis (ii) anticipated that kinetic parameters of the SOFA score and hypoxia markers would better predict death in patients with higher BOD. This hypothesis could be confirmed for SOFA and SAH, but only with limitations for lactate: First, the kinetic parameters of the SOFA score, lactate, and SAH were all significantly higher in non-survivors compared to survivors among patients with high BOD, but not in those with low BOD (Table 2). Second, the SOFA score demonstrated better discrimination for mortality in patients with high BOD compared to those with low BOD (Table 3). Surprisingly, the AUROCs of the kinetic parameters of lactate for mortality prediction showed better performance in patients with low BOD, whereas in patients with high BOD, the values were poor. Patterns of mortality prediction by kinetic SAH parameters were similar to those of the SOFA score but contrary to those of lactate, with excellent AUROC values in patients with high BOD and poor performance in patients with low BOD (Table 3). Differences in the AUROC values of the mean and the NAS of SAH between patient groups with low and high BOD showed a trend toward statistical significance (Table 3 and Figure 5). This supported a superior performance of SAH kinetic parameters for mortality prediction in patients with higher BOD. In contrast, lactate kinetic parameters did not show the expected increase in predictive power in patients with high BOD.

Hypothesis (iii), which anticipated stronger correlations between the kinetic parameters of hypoxia markers and the respective SOFA scores in patients with high BOD compared to those with low BOD, was confirmed more clearly for SAH than for lactate (Table 4, Figure 6, and Supplementary Table S1). In patients with low BOD, except for mean SAH, no further SAH parameters and none of the lactate parameters significantly correlated with the dynamic severity of organ dysfunction represented by kinetic SOFA parameters. However, in patients with high BOD, this correlation was clearly evident. Notably, correlations of the kinetic parameters of lactate with the respective SOFA scores were moderate, while those of SAH were strong. In survivors with high BOD, lactate parameters showed a significant linear correlation with the respective SOFA scores, but this was not observed in non-survivors. This result is unexpected, as a stronger correlation between the hypoxia marker and the severity of organ dysfunction would be anticipated in the more critically ill non-surviving patients. Meeting this expectation, the kinetic parameters of SAH did not correlate with the respective SOFA scores in survivors but showed a strong correlation in the subgroup of more severely ill non-survivors.

Taken together, although both lactate [7,9,40] and SAH [20,21] are supposed to be produced by cells in response to a lack of oxygen, their behavior in patients with low and high BOD revealed substantial differences in terms of mortality prediction and correlation with the severity of organ dysfunction. Assuming that hypoxic mechanisms are more active in the more severely ill patients with high BOD than in those with low BOD [4,5], a better prediction of mortality and a stronger correlation with organ dysfunction by kinetic parameters would have been expected. However, these expectations were not met by lactate but were completely fulfilled by SAH.

Considering the links between impaired microcirculation, hypoxia, organ dysfunction, and mortality [2,3,6], the findings of this study challenge the reliability of lactate as a solid hypoxia marker in patients with high BOD. Thus, the observed increases in lactate in patients with low BOD seem unlikely to be caused solely by hypoxia and might instead be due to other non-specific mechanisms. Several mechanisms of lactate production apart from hypoxia are known, such as its formation through the enhanced catecholaminergic stimulation of the glycolytic metabolism [13], the inhibition of pyruvate dehydrogenase [14], or impaired hepatic metabolization [15]. Mean lactate and lactate area scores, although statistically significant, differed only by 0.3 mmol/L and 0.4 mmol/L, respectively, in non-survivors compared to survivors in patients with low and high BOD (Table 2). Considering these minor differences between survivors and non-survivors, these values do not seem particularly useful for predicting outcomes on an individual basis in clinical practice. Moreover, the mean initial lactate concentration in non-survivors with high BOD, the most severely ill group in this study, was only 1.6 mmol/L. In contrast, previous studies demonstrating the usefulness of the kinetic parameters of lactate as predictors of in-hospital mortality showed much higher initial lactate concentrations, ranging from 3.9 to 6.16 mmol/L [11,41,42,43], clearly exceeding the threshold for diagnosing metabolic shock. Thus, in the present study, even in the group of patients with a high BOD who did not survive the hospital stay, initial and mean lactate values and their NASs were below 2 mmol/L, failing to reach clinically relevant levels for the diagnosis of shock [44].

In contrast, the kinetic parameters of SAH were more than twice as high in non-survivors compared to survivors among patients with low BOD and more than three times as high in patients with high BOD. Furthermore, the kinetic parameters of SAH were significant predictors of death with excellent discriminatory performance in patients with high BOD in univariate analyses. Additionally, SAH parameters showed a strong correlation with the severity of organ dysfunction, especially in non-survivors with high BOD.

In conclusion, the results of the present study suggest a more specific role for SAH compared to lactate in indicating hypoxia and its relation to organ dysfunction in critically ill patients. Our analyses revealed that SAH and SOFA behave analogously, while lactate deviates in its associations. Since the kinetics of SAH could mirror the kinetics of SOFA-recorded organ failure, especially in patients with high BOD, there are indications that it might be a more suitable biomarker for organ dysfunction and the underlying pathophysiological processes than lactate. Determining SAH rather than lactate in critically ill patients with a high BOD seems promising for gaining better insight into the hypoxic mechanisms of organ dysfunction and mortality. Future research is encouraged to compare markers of hypoxia and organ dysfunction in patient groups with low and high BOD.

From a clinician’s perspective, one of the most intriguing questions is the prognosis of a patient upon ICU admission which is of special interest in the most severely ill subpopulation with a high BOD. In patients with severe organ dysfunction in this study, it was SAH, not lactate, that mirrored the kinetics of organ dysfunction and enabled early mortality prediction with excellent AUROC values. If SAH is high, this might hint at ongoing microcirculatory disorders and the patient is at increased risk for organ failure and death. This could possibly trigger diagnostic efforts and enhanced hemodynamic therapy to improve tissue oxygenation, especially in patients with high BOD.

### Study Limitations

Several issues warrant critical discussion. First, this study presents secondary analyses from a prospective study aimed at identifying patients at high risk of developing sepsis [23]. Consequently, the present analyses derive from a distinct subgroup of patients, which may not represent the full range of critically ill patients typically admitted to an interdisciplinary surgical ICU yet ensured that the study population was critically ill. Second, the subpopulations with low and high BOD differed in their underlying diseases, making it unclear to what extent our results can be generalized to differently structured cohorts. However, the SOFA score has been validated in mixed medical and surgical populations of critically ill patients, encompassing a wide range of inflammatory and non-inflammatory diseases, to capture organ dysfunction and its progression for mortality prediction [25,30,31,32]. This strongly supports the existence of a common pathogenetic principle linking various disease etiologies to the clinical manifestation of multiple organ dysfunctions. In this context, microcirculatory impairment, regardless of its cause, has emerged as a universally valid pathogenetic concept for the development of multiple organ dysfunction and failure [3]. If compromised oxygen supply, resulting tissue hypoxia, and energetic failure, irrespective of their nature, lead to multiple organ dysfunction, the approach presented here should be applicable to other mixed patient populations. However, this needs to be confirmed in future studies, preferably with patient populations that are more homogeneous in terms of the etiology of their critical illness. Third, the study’s cohort was relatively small, heightening the potential for sampling bias. This issue was compounded by the formation of subgroups, which further diminished the number of patients available for statistical analyses in group comparisons. Nonetheless, both inter- and intragroup comparisons were conducted on a population delineated by prospectively defined inclusion and exclusion criteria. Although a larger patient sample could enhance statistical robustness, SOFA scores and biomarkers were repetitively monitored at 8 h intervals over up to five days. Depending on the question of interest, if this longitudinal approach—tracking the course of patients’ critical illness—was to shift to a horizontal cross-sectional design focusing on the time point of admission, the requisite patient count would be 1485 (calculated as 99 patients across 15 time points). Fourth, the optimal cutoff for categorizing patients into low and high BOD groups is unknown. Other studies with similar approaches for different research questions have used SOFA cutoff values between 5 and 10 [1,5,9,45]. We followed a similar approach to Brouwer et al. [5] by using the median initial SOFA score of the entire cohort as the cutoff. Around this value, there is not only a literature-based increase in mortality [30,31,32], but also, in our cohort, an inflection point in mortality was observed exactly at this cutoff.

## 4. Materials and Methods

### 4.1. Ethics and General Aspects

This was a secondary analysis of data from a recently published monocentric, prospective observational clinical study [23]. This study was approved by the local ethics committee (see also Institutional Review Board Statement). All patients or their legal representatives provided informed consent. After recovery, previously non-self-consenting patients had the opportunity to withdraw their consent to participate in this study. The study design and reporting were based on the recommendations of the STROBE statement (https://www.strobe-statement.org/, accessed on 3 October 2024). The study was conducted in the 24-bed ICU of the Department of Anesthesiology, Surgical Intensive Care Medicine and Pain Medicine, Mannheim University Hospital, Mannheim Medical Faculty, University of Heidelberg, from June 2017 to June 2019. Due to the initiation time of the original study, the prospective recruitment strategy was based on the sepsis-1/2 definition [46]. Accordingly, inclusion criteria for study patients were either continuous SIRS status for 48 h or sepsis on ICU admission according to sepsis-1/2 definition criteria (for further detail see [23]). The dynamics of patients’ statuses were assessed at study inclusion (referred to as time point 1) and every 8 h thereafter for up to 5 days, resulting in up to 15 evaluation time points. At each evaluation time point, the SOFA score [25] was calculated and blood was drawn for the analyses of lactate and SAH plasma concentrations. Additionally, sepsis-3 criteria [35] were applied to classify patients at evaluation time points as having no sepsis (0), sepsis (1), or septic shock (2), respectively, using an automated algorithm, as described previously [39,47]. The clinical data of ICU patients were collected using the IntelliSpace Critical Care and Anesthesia (ICCA)™ system (Philips N.V., Amsterdam, The Netherlands).

Exclusion criteria were age < 18 years, immunosuppression, end-stage renal failure, pregnancy, ECMO therapy, and a neurosurgical main diagnosis to exclude confounding neuroinflammation.

For SAH measurements, venous blood samples were obtained via a central catheter or a peripheral indwelling cannula. The laboratory staff were unaware of the patients’ conditions, while the medical specialists were blinded to the SAH measurements. Lactate levels were determined through arterial blood gas analyses using a blood gas analyzer (Radiometer ABL 800 Flex, Radiometer, Willich, Germany). SAH plasma concentrations were measured by stable-isotope dilution LC–tandem MS/MS, following the separation of analytes using pH-dependent solid-phase extraction columns containing phenylboronic acid, as previously described [23,48].

### 4.2. Calculation of Kinetic Parameters of the SOFA Score, Lactate, and SAH Plasma Concentrations

For SOFA scores, lactate, and SAH plasma concentrations kinetic parameters, i.e., the maximum, the mean, and the NAS, were determined for the total study period of up to 15 measurements within up to 5 days. The NAS was obtained by dividing the sum of the areas under the curve by the time [10,11,24]. For the SOFA score, the mean and the NAS were considered to reflect the burden of organ dysfunction. In the case of the metabolic markers, lactate, and SAH, these parameters reflected the burden of tissue hypoxia [10,11].

### 4.3. Statistical Analyses

For clinical characterization, continuous variables were compared using a t-test (Satterthwaite method), while categorical variables were assessed with the chi^2^ test. Non-normally distributed variables were compared using the Mann–Whitney U test. All variables are presented as medians (interquartile ranges (IQRs)) unless otherwise indicated. Categorical variables are expressed as numbers and percentages. Univariate logistic regression analysis was conducted to examine associations between the parameters of the SOFA score, lactate, and SAH, respectively, and in-hospital mortality. The performance of univariate logistic regression models was evaluated by the estimated coefficients and calculated odds ratios. The estimated coefficient used as an exponent to the basis of 2.7182 represents the factor by which the odds ratio of in-hospital death changes with a one-unit increase in the variable of interest. The predictive power of a variable of interest, i.e., its ability to distinguish between patients who survive and those who die, was assessed using AUROC curves. Differences in AUROCs within and between groups were calculated and tested for statistical significance. The semi-qualitative evaluation of the AUROCs’ performance followed the suggestions by Hosmer and Lemeshow [33]. For the analyses of correlations between lactate or SAH parameters and respective SOFA scores, Spearman’s rank and linear regression correlation coefficients were calculated. Except for the results in parenthesis in Figure 6, no outlier or extreme value corrections were applied, and no imputation of missing data points was performed for any directly measured or calculated variable. Despite the prediction of results according to hypotheses, all tests in this study were two-sided, and a *p*-value of less than 0.05 was considered statistically significant. In cases of multiple testing on two groups regarding the null hypothesis that the two groups are identical for a given variable in all comparisons, the level of statistical significance for rejection of the null hypothesis was corrected according to Bonferroni’s method [49]. Otherwise, Bonferroni correction was not applied due to the risk of an increase in type II errors. Statistical analyses were performed using SAS Software Version 9.4 (SAS Institute, Cary, NC, USA) or IBM SPSS Statistics Version 27 (IBM, Albany, NY, USA).

## Figures and Tables

**Table 1:** Baseline and clinical characteristics of the study population.

								Low BOD (SOFA ≤ 8)					High BOD (SOFA > 8)				
		All (N = 99)	Low BOD (N = 53)		High BOD (N = 46)			Survivors (S) (N = 42)		Non-Survivors (NS) (N = 11)			Survivors (S) (N =33)		Non-Survivors (NS) (N = 13)		
	n		n		n		Low vs. High BOD	n		n		Low BOD S vs. NS	n		n		High BOD S vs. NS
**Demographics**																	
Age (years)	99	63 (53–76)	53	67 (60–78)	46	56 (49–68)	**0.015**	42	67 (60–78)	11	76 (61–80)	0.195	33	54 (43–63)	13	68 (59–78)	**0.001**
Male (%)		65 (66)		29 (54.7)		36 (78.3)	**0.014**		21 (50.0)		8 (72.7)	0.308		25 (75.8)		11 (84.6)	0.700
**Clinical course**																	
Mechanical ventilation (%)		86 (86.9)		41 (77.4)		45 (97.8)	**0.004**		31 (73.8)		10 (90.9)	0.420		33 (100)		12 (92.3)	0.283
Vasopressor therapy (%)		68 (69)		24 (45.3)		44 (95.7)	**<0.001**		17 (40.5))		7 (63.6)	0.190		31 (93.9)		13 (100)	1.000
ICU-LOS (days)	99	25 (16–47)	53	21.3 (14.2–37.9)	46	30.7 (20.4–68.9)	**0.006**	42	23.0 (14.9–38.0)	11	18.6 (10.3–32.3)	0.232	33	36.9 (21.7–86.8)	13	23.9 (14.7–35.9)	**0.048**
In-hospital mortality (%)		24 (24)		11 (20.8)		13 (28.3)	0.385										
**Primary diagnosis (%)**																	
Major surgery		11 (11.1)		9 (17.0)		2 (4.35)	**0.046**		7 (16.7)		2 (18.2)	1.000		1 (3.0)		1 (7.7)	0.489
Sepsis		20 (20.2)		15 (28.3)		5 (10.9)	**0.031**		11 (26.2)		4 (36.4)	0.708		1 (3.0)		4 (30.8)	**0.018**
Cardiac arrest		2 (2.0)		1 (1.9)		1 (2.2)	1.000		1 (2.4)			1.000		1 (3.0)			1.000
Polytrauma		42 (42.4)		16 (30.2)		26 (56.5)	**0.008**		13 (31.0)		3 (27.3)	1.000		23 (69.7)		3 (23.1)	**0.004**
Major bleeding		14 (14.1)		4 (7.6)		10 (21.7)	**0.043**		4 (9.5)			0.569		5 (15.2)		5 (38.5)	0.117
Respiratory insuff./ARDS		10 (10.1)		8 (15.1)		2 (4.4)	0.100		6 (14.3)		2 (18.2)	0.665		2 (6.1)			1.000
**Comorbidities (%)**																	
Cardiac		35 (35.4)		24 (45.3)		11 (23.9)	**0.027**		18 (42.9)		6 (54.5)	0.518		7 (21.2)		4 (30.8)	0.702
Vascular		21 (21.2)		12 (22.6)		9 (19.6)	0.709		7 (16.7)		5 (45.5)	0.098		4 (12.1)		5 (38.5)	0.092
Arterial hypertension		51 (51.5)		34 (64.2)		17 (37.0)	**0.007**		26 (61.9)		8 (72.7)	0.726		10 (30.3)		7 (53.8)	0.181
Pulmonary		12 (12.1)		10 (18.9)		2 (4.4)	**0.027**		6 (14.3)		4 (36.4)	0.187		2 (6.1)			1.000
Renal		20 (20.2)		13 (24.5)		7 (15.2)	0.250		8 (19.0)		5 (45.5)	0.112		4 (12.1)		3 (23.1)	0.385
Hepatic		6 (6.1)		2 (3.77)		4 (8.7)	0.412		1 (2.4)		1 (9.1)	0.375		3 (9.1)		1 (7.7)	1.000
Diabetes mellitus		17 (17.2)		13 (24.5)		4 (8.7)	**0.037**		10 (23.8)		3 (27.3)	1.000		3 (9.1)		1 (7.7)	1.000
Metabolic		10 (10.1)		3 (5.7)		7 (15.2)	0.181		2 (4.8)		1 (9.1)	0.510		6 (18.2)		1 (7.7)	0.654
Cerebral		11 (11.1)		7 (13.2)		4 (8.7)	0.476		6 (14.3)		1 (9.1)	1.000		3 (9.1)		1 (7.7)	1.000
Smoking		7 (7.1)		4 (7.6)		3 (6.5)	1.000		2 (4.8)		2 (18.2)	0.186		2 (6.1)		1 (7.7)	1.000
Alcoholism		6 (6.1)		2 (3.8)		4 (8.7)	0.412				2 (18.2)	0.040		3 (9.1)		1 (7.7)	1.000
**Clinical chemistry**																	
Creatinine (mg/dL)	96	0.98 (0.73–1.50)	50	0.79 (0.61–1.22)	46	1.28 (0.89–1.83)	**0.017**	39	0.76 (0.61–1.06)	11	0.89 (0.63–2.82)	0.163	3	1.07 (0.79–1.38)	13	1.79 (1.68–2.25)	**0.008**
Urea (mg/dL)	96	45.1 (33.4–63.8)	50	44.8 (31.6–63.8)	46	45.4 (35.5–62.9)	0.990	39	41.0 (28.5–59.9)	11	56.6 (35.6–103.0)	0.106	33	43.6 (33.3–50.9)	13	62.9 (48.7–68.4)	**0.017**
Potassium (mmol/L)	98	4.1 (3.8–4.3)	52	4.0 (3.8–4.2)	46	4.1 (4.0–4.4)	0.084	41	3.9 (3.7–4.2)	11	4.3 (4.1–4.5)	0.053	33	4.1 (4.0–4.3)	13	4.2 (4.0–4.5)	0.423
Bilirubin (mg/dL)	93	0.61 (0.35–0.94)	49	0.45 (0.32–0.69)	44	0.84 (0.51–1.30)	**0.006**	39	0.45 (0.32–0.76)	10	0.48 (0.37–0.56)	0.785	32	0.77 (0.51–1.28)	12	0.90 (0.61–1.42)	0.496
AST (U/L)	90	43 (27–90)	49	36 (24–52)	41	75 (42–181)	**<0.001**	39	37 (24–54)	10	28 (19–48)	0.323	30	78 (42–182)	11	70 (29–111)	0.955
ALT (U/L)	92	37 (20–85)	49	26 (19–45)	43	55 (30–199)	**0.001**	39	35 (17–47)	10	21 (19–26)	0.309	31	61 (32–206)	12	38 (28–158)	0.953
Lipase (U/L)	90	86 (61–207)	47	80 (50–146)	43	104 (64–357)	**0.031**	38	83 (50–146)	9	66 (49–82)	0.351	31	94 (63–297)	12	201 (72–541)	0.357
CRP (mg/dL)	96	150 (90–218)	50	140 (87–215)	46	165 (90–223)	0.577	39	148 (85–215)	11	114 (87–218)	0.761	33	175 (92–223)	13	155 (81–214)	0.678
PCT (µg/L)	69	0.63 (0.20–2.29)	36	0.30 (0.15–0.87)	33	1.24 (0.40–3.00)	**0.003**	28	0.26 (0.12–0.62)	8	0.52 (0.27–1.20)	0.304	24	1.46 (0.52–2.81)	9	0.99 (0.40–7.04)	0.952
**Hematology**																	
Hemoglobin (g/dL)	98	8.85 (8.10–10.1)	52	8.90 (8.20–10.6)	46	8.80 (7.80–9.60)	**0.048**	41	8.90 (8.10–10.8)	11	8.90 (8.30–9.70)	0.631	33	8.80 (7.80–9.60)	13	8.80 (8.50–9.50)	0.543
WBC (10^9^/L)	96	11.5 (8.3–14.3)	50	12.5 (10.3–15.4)	46	9.4 (6.3–13.0)	**0.005**	39	8.9 (8.1–10.8)	11	12.6 (7.5–13.8)	0.573	33	9.9 (7.9–12.8)	13	7.3 (4.8–14.9)	0.510
Thrombocytes (10^9^/L)	96	162 (106–250)	50	203 (142–275)	46	123 (82–195)	**<0.001**	39	198 (142–279)	11	229 (136–252)	0.963	33	133 (84–195)	13	103 (81–138)	0.386
INR	96	1.07 (1.00–1.12)	50	1.04 (0.99–1.10)	46	1.10 (1.04–1.19)	**0.003**	39	1.04 (1.00–1.10)	11	1.01 (0.95–1.12)	0.606	33	1.07 (1.02–1.16)	13	1.12 (1.07–1.19)	0.271
**Vital signs**																	
Temperature (°C)	96	37.1 (36.8–37.7)	50	37.0 (36.7–37.5)	46	37.2 (36.8–37.8)	0.157	39	37.0 (36.6–37.6)	11	37.1 (36.7–37.5)	0.892	33	37.3 (36.9–37.8)	13	37.1 (36.8–38.0)	0.817
Respiratory rate (1/min)	98	19 (16–22)	52	18 (16–22)	46	20 (16–23)	0.390	41	18 (16–21)	11	20 (16–27)	0.224	33	20 (18–22)	13	19 (16–24)	0.985
Horovitz index (mmHg)	98	280 (220–343)	52	295 (224–353)	46	271 (214–326)	0.276	41	302 (234–357)	11	215 (183–295)	0.271	33	286 (227–326)	13	220 (188–300)	0.294
Shock index	98	0.70 (0.61–0.88)	52	0.66 (0.54–0.82)	46	0.80 (0.65–0.93)	**<0.001**	41	0.66 (0.55–0.82)	11	0.62 (0.49–0.83)	0.856	33	0.80 (0.66–0.89)	13	0.76 (0.64–1.01)	0.495
**Clinical scores**																	
RASS	97	−3 (−5–0)	51	−1 (−3–0)	46	−5 (−5–−3)	**<0.001**	41	−1 (−3–0)	10	0 (−1–1)	0.084	33	−5 (−5–−3)	13	−4 (−5–−1)	0.423
TISS	98	18 (10–22)	52	12 (10–18)	46	22 (18–23)	**<0.001**	41	10 (10–18)	11	13 (10–18)	0.386	33	22 (18–23)	13	23 (22–27)	**0.028**
SAPS II	98	35 (28–43)	52	35 (29–42)	46	35 (26–45)	0.975	41	10 (10–18)	11	39 (35–45)	0.110	33	30 (23–38)	13	45 (40–51)	**<0.001**
SOFA	99	8 (5–11)	53	6 (4–7)	46	12 (10–13)	**<0.001**	42	5 (4–7)	11	6 (5–8)	**0.040**	33	11 (10–12)	13	13 (12–15)	**0.003**
**Hypoxia biomarkers**																	
Lactate (mmol/L)	98	1.0 (0.7–1.6)	52	0.9 (0.6–1.1)	46	1.3 (0.8–1.9)	**0.004**	41	0.8 (0.6–1.2)	11	1.1 (0.6–1.1)	0.543	33	1 (0.8–1.5)	13	1.6 (1.4–3.0)	**0.031**
SAH (nmol/L)	95	21.7 (13.5–47.8)	53	18.6 (11.6–32.0)	46	30.5 (16.5–59.8)	**0.018**	42	18.4 (11.5–29.0)	11	24.7 (11.6–101.0)	0.308	33	19.3 (14.8–41.3)	13	49.8 (44.8–85.3)	**0.001**

Results are given as median (interquartile range, IQR) or number (%); significant results are highlighted in bold. Abbreviations: BOD = burden of organ dysfunction; N = number of patients included; n = number of determinations for the variable; insuff. = insufficiency; ARDS = acute respiratory distress syndrome; ICU = intensive care unit; LOS = length of stay; AST = aspartate aminotransferase; ALT = alanine aminotransferase; CRP = C-reactive protein; PCT = procalcitonin; WBC = white blood cell; INR = international normalized ratio; RASS = Richmond Agitation and Sedation Score; TISS = Therapeutic Intervention Severity Score; SAPS = Simplified Acute Physiology Score; SOFA = Sequential Organ Failure Assessment score.

**Table 2:** Initial values and kinetic parameters of serially determined SOFA scores, lactate, and SAH plasma concentrations grouped by burden of organ dysfunction (BOD) and mortality.

Low BOD (SOFA ≤ 8)					High BOD (SOFA > 8)				
	All (N = 53)	Survivors (S) (N = 42)	Non-Survivors (NS) (N = 11)	S vs. NS	All (N = 46)	Survivors (N = 33)	Non-Survivors (N = 13)	S vs. NS	Low BOD vs. High BOD
**SOFA**									
Initial/n	6.0 (4.0–7.0)/53	5.0 (4.0–7.0)/42	6.0 (5.0–8.0)/11	**0.038**	12.0 (10.0–13.0)/46	11.0 (9.5–12.5)/33	13.0 (12.0–15.0)/13	**0.003**	**<0.001**
Maximum/n	7.0 (5.0–8.0)/53	6.0 (4.8–8.0)/42	7.0 (6.0–8.0)/11	0.058	12.0 (10.0–14.0)/46	11.0 (10.0–12.5)/33	15.0 (12.0–15.5)/13	**<0.001**	**<0.001**
Mean/n	4.9 (3.2–6.0)/522	4.4 (2.8–5.8)/406	5.7 (5.1–7.2)/116	**0.014**	8.9 (6.7–11.3)/621	8.0 (6.3–10.0)/469	12.7 (9.5–14.8)/152	**<0.001**	**<0.001**
NAS/n	4.8 (3.4–5.9)/522	4.4 (2.8–5.9)/406	5.6 (4.8–7.3)/116	**0.025**	8.8 (6.6–11.2)/621	8.1 (6.2–9.9)/469	12.7 (9.5–1.8)/152	**<0.001**	**<0.001**
**Lactate (mmol/L)**									
Initial/n	0.9 (0.6–1.1)/52	0.8 (0.6–1.2)/41	1.1 (0.6–1.1)/11	0.535	1.3 (0.8–2.0)/46	1.0 (0.8–1.7)/33	1.6 (1.2–3.2)/13	**0.030**	**0.004**
Maximum/n	1.2 (1.0–1.8)/51	1.2 (0.9–1.8)/41	1.8 (1.3–2.6)/10	**0.027**	1.6 (1.3–2.5)/46	1.5 (1.2–2.1)/33	2.3 (1.4–3.2/13	**0.049**	**<0.001**
Mean/n	0.9 (0.7–1.2)/506	0.9 (0.7–1.1)/389	1.2 (1.0–1.5)/117	**0.003**	1.1 (0.9–1.5)/620	1.0 (0.9–1.3)/470	1.4 (1.0–1.8)/150	**0.047**	**0.005**
NAS/n	1.0 (0.7–1.2)/506	0.9 (0.7–1.1)/389	1.2 (1.0–1.5)/117	**0.004**	1.1 (0.9–1.4)/620	1.0 (0.8–1.3)/470	1.4 (0.9–1.8)/150	**0.039**	**0.008**
**SAH (nmol/L)**									
Initial/n	18.6 (11.6–32.8)/53	18.4 (11.5–29.2)/42	24.7 (11.6–101.1)/11	0.303	30.5 (16.5–59.9)/46	19.3 (14.2–42.1)/33	49.8 (44.0–87.0)/13	**<0.001**	**0.018**
Maximum/n	25.2 (17.4–48.3)/53	24.1 (15.9–42.8)/42	49.6 (19.7–143.8)/11	0.076	46.5 (28.6–98.5)/46	35.4 (26.7–60.4)/33	115.0 (49.7–194.1)/13	**<0.001**	**<0.001**
Mean/n	18.9 (12.7–36.5)/609	18.6 (12.0–30.0)/467	40.9 (12.8–116.4)/142	0.177	27.8 (20.2–65.2)/639	24.3 (18.1–36.3)/480	79.1 (35.2–127.0)/159	**<0.001**	**0.002**
NAS/n	18.5 (12.3–36.6)/609	18.2 (11.9–30.1)/467	40.7 (12.6–118.1)/142	0.188	27.6 (20.4–65.6)/639	24.6 (17.6–36.2)/480	81.3 (35.0–127.0)/159	**<0.001**	**0.002**

The initial values refer to the determination and measurements of the SOFA score and blood sampling, respectively, at the time of the patients’ study inclusion. The maximum and mean values represent the highest and the average calculated from all values taken across up to 15 time points, with assessments conducted every 8 h, covering a span of up to 112 h within the total study period. Normalized area scores (NAS) were obtained by plotting the values of the SOFA score, lactate, and SAH for each patient over the designated timeframe. These were then calculated by dividing the areas under the curve by the period of observation. For further details, please refer to Section 4.2 within Materials and Methods. Results are given as the median (interquartile range, IQR). Significant differences between survivors and non-survivors or between low and high BOD groups are highlighted in bold. Abbreviations: N = number of patients included; n = number of determinations for each parameter; SOFA = Sequential Organ Failure Assessment. Lactate and SAH refer to plasma concentrations of lactate and S-adenosylhomocysteine.

**Table 3:** Univariate logistic regression analyses of initial values and kinetic parameters of serially determined SOFA scores, lactate, and S-adenosylhomocysteine plasma concentrations for the prediction of in-hospital mortality in patients grouped by burden of organ dysfunction (BOD).

	Low BOD (SOFA ≤ 8)					High BOD (SOFA > 8)						
	Coefficient (Means, SE)	Odds Ratio (95% CI)	*p*-Value	AUROC (SE)	*p*-Value	Coefficient (Means, SE)	Odds Ratio (95% CI)	*p*-Value	AUROC (SE)	*p*-Value	∆ AUROC = AUROC_High BOD_ − AUROC_Low BOD_	*p*-Value
**SOFA**												
Initial	0.476 (0.239)	1.609 (1.007–2.571)	**0.047**	**0.701 (0.079)**	**0.041**	0.537 (0.196)	1.711 (1.165–2.515)	**0.006**	**0.780 (0.071)**	**0.003**	0.078 (0.106)	0.460
Maximum	0.276 (0.144)	1.318 (0.994–1.749)	0.055	0.685 (0.076)	0.061	0.603 (0.197)	1.828 (1.241–2.691)	**0.002**	**0.815 (0.072)**	**0.001**	0.130 (0.105)	0.217
Mean	0.178 (0.126)	1.195 (0.934–1.529)	0.157	**0.741 (0.070)**	**0.014**	0.541 (0.172)	1.718 (1.228–2.405)	**0.002**	**0.838 (0.066)**	**<0.001**	0.097 (0.096)	0.313
NAS	0.327 (0.169)	1.386 (0.996–1.929)	0.053	**0.723 (0.073)**	**0.024**	0.540 (0.172)	1.717 (1.226–2.403)	**0.002**	**0.840 (0.065)**	**<0.001**	0.117 (0.098)	0.229
**Lactate**												
Initial	−0.116 (0.769)	0.890 (0.197–4.018)	0.880	0.561 (0.092)	0.538	0.517 (0.299)	1.678 (0.933–3.016)	0.084	**0.706 (0.086)**	**0.031**	0.145 (0.126)	0.248
Maximum	1.552 (0.645)	4.721 (1.334–16.71)	**0.016**	**0.718 (0.090)**	**0.027**	0.593 (0.358)	1.810 (0.897–3.653)	0.98	0.688 (0.091)	**0.050**	−0.031 (0.128)	0.811
Mean	4.085 (1.387)	59.46 (3.924–901.3)	**0.003**	0.789 (0.082) *	**0.003**	1.660 (0.877)	5.262 (0.943–29.349)	0.058	0.689 (0.093)	**0.048**	−0.101 (0.124)	0.417
NAS	3.921 (1.382)	50.46 (3.365–756.9)	**0.005**	0.789 (0.082) *	**0.004**	1.728 (0.885)	5.686 (1.004–32.194)	**0.049**	0.697 (0.095)	**0.039**	−0.092 (0.124)	0.461
**SAH**												
Initial	0.025 (0.011)	1.025 (1.004–1.047)	**0.019**	0.602 (0.118)	0.303	0.030 (0.110)	1.030 (1.008–1.053)	**0.009**	**0.825 (0.061)**	**0.001**	0.223 (0.132)	0.091
Maximum	0.010 (0.006)	1.010 (0.999–1.021)	0.088	0.675 (0.100)	0.076	0.026 (0.008)	1.026 (1.010–1.043)	**0.002**	**0.837 (0.066)**	**<0.001**	0.162 (0.120)	0.177
Mean	0.023 (0.009)	1.023 (1.005–1.041)	**0.012**	0.633 (0.111)	0.177	0.039 (0.120)	1.039 (1.015–1.065)	**0.002**	**0.865 (0.057)**	**<0.001**	0.232 (0.125)	0.064
NAS	0.018 (0.008)	1.018 (1.002–1.034)	**0.026**	0.630 (0.111)	0.188	0.039 (0.012)	1.040 (1.015–1.065)	**0.002**	**0.867 (0.056)**	**<0.001**	0.237 (0.124)	0.056

Models were calculated using univariate logistic regression (for further details see Section 2.5 and Section 4.3 in Materials and Methods). The regression coefficient, odds ratio, and AUROCs are presented as means with standard errors (SE) or within a 95% confidence interval (CI). Significant *p*-values are highlighted in bold. AUROC values are highlighted in bold only if both the logistic regression and AUROC analysis reached the level of statistical significance, and the AUROC value was at least acceptable (AUROC 0.7–0.8) or excellent (AUROC 0.8–0.9) according to the classification by Hosmer and Lemeshow [33]. AUROCs between the initial and kinetic parameters for lactate differed significantly (* *p* = 0.003 for mean lactate and lactate NAS). Intergroup differences in AUROCs were calculated as ∆ AUROC = AUROC_High BOD_ − AUROC_Low BOD_. The calculated intergroup differences in AUROCs showed a trend toward statistical significance for the mean and NAS values of SAH. Abbreviations: SOFA = Sequential Organ Failure Assessment; AUROC = area under the receiver operating characteristic curve; NAS = normalized area score. For further explanation of initial values and kinetic parameters (maximum, mean, and NAS), see the legend of Table 2.

**Table 4:** Linear regression correlation matrix for the initial values and kinetic parameters of lactate and SAH with corresponding SOFA parameters in patients grouped by burden of organ dysfunction (BOD) and mortality.

	Low BOD (SOFA ≤ 8) (N = 53)	High BOD (SOFA > 8) (N = 46)
	Linear Regression (r/*p*/n/a)	Linear Regression (r/*p*/n/a)
	**All** (N = 53)	**All** (N = 46)
**Initial**	Initial SOFA	
Lactate	0.114/0.414/52/1	0.487/**<0.001/**46/1
SAH	0.071/0.621/53/1	**0.615/<0.001/**46/1
**Maximum**	Maximum SOFA	
Lactate	0.202/0.148/52/1	0.489/**<0.001/**46/1
SAH	0.158/0.254/53/1	**0.629/<0.001/**46/1
**Mean**	Mean SOFA	
Lactate	0.044/0.740/52/9.5	0.493/**<0.001/**46/13.5
SAH	0.170/0.220/53/9.8	**0.684/<0.001/**46/13.5
**NAS**	NAS of SOFA	
Lactate	0.148/0.300/51/9.5	0.495/**<0.001/**46/13.5
SAH	0.176/0.208/53/9.8	**0.678/<0.001/**46/13.5
	**Survivors** (N = 42)	**Survivors** (N = 33)
**Initial**	Initial SOFA	
Lactate	0.077/0.631/41/1	0.436/**0.011/**33/1
SAH	0.167/0.287/42/1	0.420/**0.015/**33/1
**Maximum**	Maximum SOFA	
Lactate	0.031/0.845 /41/1	0.388/**0.026/**33/1
SAH	0.077/0.614/42/1	0.281/0.113/33/1
**Mean**	Mean SOFA	
Lactate	0.151/0.345/41/9.2	0.368/**0.035/**33/14.2
SAH	0.095/0.548/42/9.7	0.170/0.343/33/14.2
**NAS**	NAS of SOFA	
Lactate	0.070/0.657/40/9.2	0.352/**0.045/**33/14.2
SAH	0.031/0.852/42/9.7	0.161/0.370/33/14.2
	**Non-survivors** (N = 11)	**Non-survivors** (N = 13)
**Initial**	Initial SOFA	
Lactate	0.437/0.179/11/1	0.427/0.145/13/1
SAH	0.192/0.178/11/1	**0.714/0.006/**13/1
**Maximum**	Maximum SOFA	
Lactate	0.246/0.463/11/1	0.464/0.110/13/1
SAH	0.011/0.752/11/1	**0.628/0.021/**13/1
**Mean**	Mean SOFA	
Lactate	0.411/0.210/11/10.5	0.522/0.067/13/11.5
SAH	0.176/0.603/11/10.5	**0.861/<0.001/**13/11.7
**NAS**	NAS of SOFA	
Lactate	0.451/0.164/11/10.5	0.524/0.066/13/11.5
SAH	0.252/0.453/11/10.5	**0.852/<0.001/**13/11.7

Significant *p*-values are highlighted in bold. Correlation coefficients are highlighted in bold if statistical significance and a strong correlation according to Cohen et al. [34] (=a linear regression coefficient r > 0.5) was reached. N = number of patients per group; n = number of variable pairs used for the calculation of the respective correlation. Since each variable pair was obtained from an individual patient, n equals N, unless values are missing. a = average number of measurements per patient used to determine the individual maximum, mean, or NAS value; SOFA = Sequential Organ Failure Assessment; NAS = normalized area score; SAH = S-adenosylhomocysteine.

## Data Availability

The data presented in this study are available on request from the corresponding author due to patient privacy.

## Supplementary Materials

The supporting information can be downloaded at: https://www.mdpi.com/article/10.3390/ijms26020659/s1.
