# Supplementary material for: Evaluation of Hypoxia Markers in Critically Ill Patients Categorized by Their Burden of Organ Dysfunction: A Novel Approach to Detect Pathophysiological and Clinical Relevance in a Secondary Analysis of a Prospective Observational Study
Source: Int J Mol Sci. 2025 Jan 14;26(2):659. doi: 10.3390/ijms26020659 (PMC11766418; doi:10.3390/ijms26020659)
Supplement: Supplementary file 1 [file ijms-26-00659-s001.zip › ijms-3271529-supplementary.pdf]

Supplementary Table S1. Spearman’s rank correlation matrix for initial values and kinetic parameters of lactate and SAH with corresponding SOFA parameters in patients grouped by burden of organ dysfunction (BOD) and mortality.

	**Low BOD (SOFA ≤ 8)** (N = 53)	**High BOD (SOFA > 8)** (N = 46)
	Spearman (r / p / n / a)	Spearman (r/p/n/a)
	**All** (N = 53)	**All** (N = 46)
**Initial**	Initial SOFA	
Lactate	-0.048 / 0.734 / 52 / 1	0.495 / **<0.001** / 46 / 1
SAH	0.140 / 0.317 / 53 / 1	**0.634 / <0.001** / 46 / 1
**Maximum**	Maximum SOFA	
Lactate	0.155 / 0.272 / 52 / 1	0.376 / **0.010** / 46 / 1
SAH	0.223 / 0.109 / 53 / 1	**0.555 / <0.001** / 46 / 1
**Mean**	Mean SOFA	
Lactate	0.211 / 0.133 / 52 / 9.5	0.466 / **0.001** / 46 / 13.5
SAH	0.310 / **0.024** / 53 / 9.8	**0.538** / **<0.001** / 46 / 13.5
**N. area score**	Normalized SOFA area score	
Lactate	0.180 / 0.207 / 51 / 9.5	0.447 / **0.002** / 46 / 13.5
SAH	0.225 / 0.105 / 52 / 9.8	**0.539** / **<0.001** / 46 / 13.5
	**Survivors** (N = 42)	**Survivors** (N = 33)
**Initial**	Initial SOFA	
Lactate	0.017 / 0.916 / 41 / 1	0.359 / **0.040** / 33 / 1
SAH	0.210 / 0.182 / 42 / 1	0.498 / **0.003** / 33 / 1
**Maximum**	Maximum SOFA	
Lactate	0.104 / 0.517 / 41 / 1	0.239 / 0.180 / 33 / 1
SAH	0.181/0.251/42/1	0.296 / 0.095 / 33 / 1
**Mean**	Mean SOFA	
Lactate	0.033 / 0.836 / 41 / 9.2	0.297 / 0.093 / 33 / 14.2
SAH	0.278 / 0.075 / 42 / 9.7	0.234 / 0.190 / 33 / 14.2
**N. area score**	Normalized SOFA area score	
Lactate	0.013 / 0.935 / 40 / 9.2	0.272 / 0.126 / 33 / 14.2
SAH	0.176 / 0.266 / 42 / 9.7	0.237 / 0.183 / 33 / 14.2
	**Non-survivors** (N = 11)	**Non-survivors** (N = 13)
**Initial**	Initial SOFA	
Lactate	-0.442 / 0.173 / 11 / 1	0.429 / 0.143 / 13 / 1
SAH	-0.275 / 0.413 / 11 / 1	0.528 / 0.064 / 13 / 1
**Maximum**	Maximum SOFA	
Lactate	0.145 / 0.670 / 11 / 1	0.394 / 0.182 / 13 / 1
SAH	0.019 / 0.956 / 11 / 1	**0.630** / **0.021** / 13 / 1
**Mean**	Mean SOFA	
Lactate	0.310 / 0.354 / 11 / 10.5	0.472 / 0.104 / 13 / 11.5
SAH	0.260 / 0.441 / 11 / 10.5	**0.853** / **<0.001** / 13 / 11.7
**N. area score**	Normalized SOFA area score	
Lactate	0.309 / 0.355 / 11 / 10.5	0.500 / 0.082 / 13 / 11.5
SAH	0.282 / 0.401 / 11 / 10.5	**0.802** / **<0.001** / 13 / 11.7

Significant p-values are highlighted in bold. Correlation coefficients are highlighted in bold if statistical significance and a strong correlation according to Cohen et al. [34] (= a Spearman correlation coefficient r > 0.5) was reached. N = number of patients per group; n = number of variable pairs used for calculation of the respective correlation. Since each variable pair is obtained from an individual patient, n equals N, unless values are missing. a = average number of measurements per patient used to determine the individual maximum, mean or normalized area score value. SOFA = Sequential Organ Failure Assessment; SAH = S-adenosylhomocysteine
